# Vulvar and Vaginal Melanomas—The Darker Shades of Gynecological Cancers

**DOI:** 10.3390/biomedicines9070758

**Published:** 2021-06-30

**Authors:** Elena-Codruța Dobrică, Cristina Vâjâitu, Carmen Elena Condrat, Dragoș Crețoiu, Ileana Popa, Bogdan Severus Gaspar, Nicolae Suciu, Sanda Maria Crețoiu, Valentin Nicolae Varlas

**Affiliations:** 1 Department of Pathophysiology, University of Medicine and Pharmacy of Craiova, 200349 Craiova, Romania; codrutadobrica@gmail.com; 2 Department of Dermatology and Allergology, “Elias” Emergency University Hospital, 011461 Bucharest, Romania; cristina.vajaitu@gmail.com; 3 Dermatology Department, Carol Davila University of Medicine and Pharmacy, 050474 Bucharest, Romania; 4 Alessandrescu-Rusescu National Institute for Mother and Child Health, Fetal Medicine Excellence Research Center, 11062 Bucharest, Romania; drcarmencondrat@gmail.com (C.E.C.); dragos.cretoiu@umfcd.ro (D.C.); nsuciu54@yahoo.com (N.S.); 5 Department of Cell and Molecular Biology and Histology, Carol Davila University of Medicine and Pharmacy, 050474 Bucharest, Romania; 6 Department of Anatomopathology, Colțea Clinical Hospital, 030167 Bucharest, Romania; ileanapopa2004@gmail.com; 7 Surgery Department, Carol Davila University of Medicine and Pharmacy, 050474 Bucharest, Romania; bogdan.gaspar@umfcd.ro; 8 Surgery Clinic, Bucharest Emergency Clinical Hospital, 014461 Bucharest, Romania; 9 Division of Obstetrics, Gynecology and Neonatology, Carol Davila University of Medicine and Pharmacy, 050474 Bucharest, Romania; 10 Department of Obstetrics and Gynecology, Filantropia Clinical Hospital, 011132 Bucharest, Romania; valentin.varlas@umfcd.ro; 11 Faculty of Dental Medicine, Carol Davila University of Medicine and Pharmacy, 050474 Bucharest, Romania

**Keywords:** vulvar melanoma, vaginal melanoma, targeted therapy, gynecological cancer, melanoma treatment

## Abstract

Melanomas of the skin are poorly circumscribed lesions, very frequently asymptomatic but unfortunately with a continuous growing incidence. In this landscape, one can distinguish melanomas originating in the mucous membranes and located in areas not exposed to the sun, namely the vulvo-vaginal melanomas. By contrast with cutaneous melanomas, the incidence of these types of melanomas is constant, being diagnosed in females in their late sixties. While hairy skin and glabrous skin melanomas of the vulva account for 5% of all cancers located in the vulva, melanomas of the vagina and urethra are particularly rare conditions. The location in areas less accessible to periodic inspection determines their diagnosis in advanced stages, often metastatic. Moreover, despite the large number of drugs newly approved in recent decades for the treatment of cutaneous melanoma, especially in the category of biological drugs, the mortality of vulvo-vaginal melanomas has remained almost constant. This, together with the absence of specific treatment guidelines due to the lack of a sufficient number of cases to conduct randomized clinical trials, makes melanomas with this localization a discouraging diagnosis, associated with a very poor prognosis. Our aim is therefore to draw attention to this oftentimes overlooked entity in order to encourage the community to employ various strategies meant to increase research in this area. By highlighting the main risk factors of vulvar and vaginal melanomas, as well as the clinical manifestations and molecular changes underlying these neoplasms, ideally novel therapeutic schemes will, in time, be brought into effect.

## 1. Introduction

Melanoma is an extremely aggressive tumor with a high metastatic rate, whose diagnosis in advanced stages was associated, until a decade ago, with minimal chances of survival [1]. It is a tumor originating in melanocyte cells which are formed during embryogenesis from the neural crest of the trunk [2]. Melanocytes come from progenitor cells with a high migration capacity. This migratory capacity explains why they are distributed and present at the level of a large number of structures: the skin—the basal layer of the epidermis (with an important role in the uniform pigmentation by forming epidermal–melanin units)—the inner ear, gastrointestinal tract and the nerve structures [3]. The incidence of melanoma has seen a spectacular increase in the last two decades, with over 300,000 new cases in 2018, the most affected countries being Australia and New Zealand (over 33 cases/100,000 inhabitants), the average age of onset of melanoma being 65 years old [4,5]. Regarding the distribution according to sex, in the case of melanomas discovered in adulthood, there is a predominance of cases in males, with a reversal of the phenomenon for melanomas diagnosed between 15–39 years, over 60% of cases appearing in the female population [6,7]. An interesting phenomenon was observed in the young population diagnosed with melanoma, the cases experiencing an alarming increase between 1999–2006, with a decrease in the next 10 years, dynamics overlapping with the increasing popularity of photoprotection methods and awareness of the danger posed by tanning beds exposure [6,8]. Due to the poorly associated prognosis determined mainly by the discovery of melanoma in advanced stages, melanoma remains the skin cancer with the highest mortality, the 5-year survival rate being less than 80% and depending on the degree of local tumor extension, lymphatic invasion, and presence of metastases [9,10]. Although dermatoscopic evaluation has become essential in pigmentary lesions screening, the diagnosis of melanoma is difficult even for experienced dermatologists, the clinical and dermatoscopic appearance presenting a great variability and sometimes even possessing misleading histological aspects that can lead to false-negative results [9,11,12].

Melanomas with genital location are included in the category of rare neoplasms, vulvar and vaginal melanomas totaling less than 1–2% of all melanomas diagnosed in females [13,14]. Vulvar melanoma represents approximately 5% of all cancers located in the vulva, being more common in adulthood (average age is 68 years) [15,16]. Between 2.5 and 4.5 patients/100,000 inhabitants are affected each year by vulvar cancer, melanoma with this location being among the top four most common vulvar cancers, the most frequent being squamous cell carcinoma (over 75% of cases versus 5.6% for melanoma) [17,18].

On the other hand, vaginal melanoma is even a rarer condition, accounting for less than 0.05–0.1% of genital neoplasms [19]. The average age of onset is lower than in the case of vulvar melanoma (57 years), and the prognosis is much worse, less than a third of patients surviving 5 years after diagnosis, despite the correct instituted treatment [20]. In a study performed on 1400 patients with vulvar melanoma and 463 with vaginal melanoma, Wohlmuth et al. highlight the occurrence of the former at a significantly younger age (*p* < 0.001) [21].

Unlike cutaneous melanoma, which is much more common in Caucasians, the frequency of vulvar and vaginal melanomas has very little variability related to race, but there is a slight increase in frequency in Whites (3.14 vs. 1.02: 1) [22]. This increase is statistically significant for vulvar melanoma, but not for those with vaginal localization (*p* < 0.001) [21].

Particular attention has been paid in the last years to the necessity of avoiding sun exposure using screen protective agents, periodic self-examination of pigmented lesions and periodic dermatoscopic surveillance of potentially evolving lesions, these being considered the most important methods to decrease the incidence and mortality of melanoma [23]. However, the genital area is often overlooked as a possible site of melanoma and other skin cancers, both by patients and doctors. Yet, melanoma with localization in the vulva and vagina is characterized by increased severity especially due to late diagnosis, most often in metastatic stages [18]. Moreover, the therapeutic means used in cutaneous melanomas are of little use in the treatment of melanomas with vulvar and vaginal localization, mortality having high values and not changing considerably in the last three decades (5-year survival rates vary between 10% and 63%) [17,24,25,26]. Thus, the present review aims to highlight the main risk factors identified in the occurrence of vulvar and vaginal melanomas, clinical manifestations, molecular changes underlying these neoplasms, as well as the main therapeutic means and their effectiveness in terms of survival.

## 2. Risk Factors

Melanoma, like most neoplastic pathologies, is a multifactorial disease, its occurrence being related to interactions between environmental and host factors, thus appearing as a consequence of genetic and epigenetic modifications that eventually lead to alteration of regulatory processes [27]. Although a number of risk factors involved in the etiopathogenesis of the disease have been identified for cutaneous melanoma, such as intermittent exposure with increased intensity to ultraviolet (UV) radiation, history of sunburns (more common at high latitudes), the presence of atypical nevi, specific skin phenotype (lightly pigmented skin, with light eyes, blond or reddish hair, presence of freckles), family history with specific genetic changes that alter the ultraviolets repair of melanocytes subjected to UV radiation, the use of Psoralen and Ultraviolet A therapy (PUVA) for a long time (over 15 years after exposure), the etiopathogenic mechanisms for vulvar and vaginal melanomas are not yet elucidated, and no specific factors involved in their occurrence have been identified [17,28,29,30,31,32]. Moreover, in a study published by Heinzelmann-Schwartzet et al., the onset of vulvar melanoma was regarded as spontaneous, de novo, with changes in a single melanocyte cell being enough to trigger the process of oncogenesis and determine the formation of melanoma [17].

However, there are studies that show a greater association between mucosal melanomas in general, and vaginal and vulvar melanomas in particular, and certain factors:

Sex. The female gender appears to be a risk factor for mucosal melanomas in general, which are twice as common as in men, compared to cutaneous melanomas whose distribution is similar between the two sexes [33].

Age. Vulvar melanoma is a disease that has an average age of onset of 68 years old, the risk of onset increasing with age (the number of cases increases from 0.11/1 million inhabitants for 15–29 years range, to 3.5/1 million inhabitants for those over 60 years old [34,35]). In contrast, cutaneous melanoma has a maximum incidence around the fourth decade of life [35].

Family history of cutaneous melanoma appears to be a risk factor for vulvar melanoma [36].

Ethnicity also seems to play an important role in the evolution of melanomas with genital localization and in their prognosis. Even if the association is not as strong as in the case of cutaneous melanomas, vulvar and vaginal melanomas are three times more common in the white race (*p* < 0.001) [22]. However, the prognosis has an inverse association, the mortality being much higher among the African population [37].

Lichen sclerosus is a precursor lesion of squamous cell carcinoma of the genital area which appears in its evolution in a percentage of approximately 5%, the causal link between the two pathologies being well known. Regarding the occurrence of vulvar melanoma in patients with lichen sclerosus, while a causal link has not been clearly established, although the number of reported cases is quite small, an increased incidence of vulvar melanoma has been observed among these patients (relative risk = 341) [38,39].

UV radiation. Though vulvar melanoma does not occur in photo-exposed areas, some studies suggest the indirect involvement of UV radiation in the pathogenesis of vulvar melanoma, through alterations of the immune response that favor modifications of the pathways involved in oncogenesis. However, it is important to note that the role of radiation in the pathogenesis of the disease is significantly lower than in skin melanoma [34].

Although the Human Papilloma Virus (HPV) is known for its roles in the development of benign (condyloma acuminata) and malignant tumors (invasive squamous cell carcinoma, anorectal cancers, penile cancers, squamous vaginal cancers) with genital localization, there is yet no mechanism to demonstrate its involvement in melanoma. There is no evidence that the HPV infection elevates the risk of vulvar or vaginal melanomas [32,40,41,42].

## 3. Clinical Manifestations

**Figure 1:**
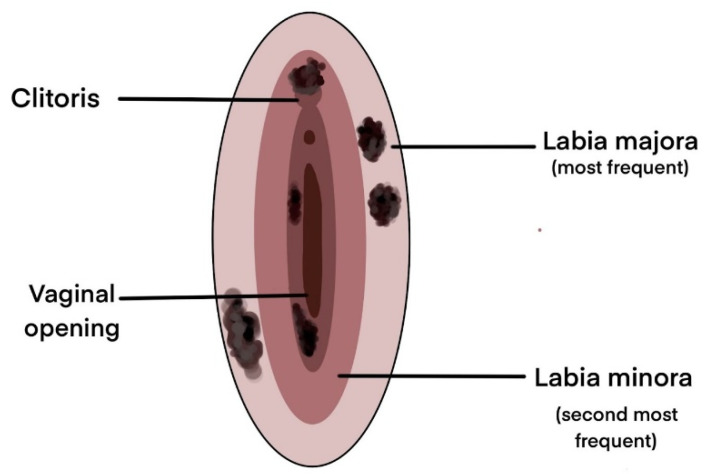
Most common sites for vulvar melanomas [15,36,47,57,58,59].

Unlike cutaneous melanoma which is most frequently distributed on photo-exposed areas (face, trunk, lower limbs) making it possible to diagnose it from early stages, vulvar and vaginal melanomas have origins that are generally overlooked by frequent inspection, the anatomical position of the lesion being the main reason for its late diagnosis and poor prognosis [43]. Melanomas of the vulvar region can occur in a variety of sites starting from the hairy skin of the labium majus to the introitus (Figure 1). The most common sites are the clitoris, the labia majora, and the labia minora, and in most cases, multiple tumors can be detected [34,44,45].

Vulvar and vaginal melanomas are pigmented lesions with variable diameters (generally over 7 mm), with macular (most frequently), papular or nodular appearance, most often asymmetrical and with an inhomogeneous appearance in terms of pigmentation [44,46,47]. There are also reports in the literature of cases with amelanotic manifestations of genital melanomas, especially at the vaginal level [20,48]. The first case of genital melanoma was described in 1861 in a 35-year-old patient who presented with multiple pigmentary lesions and evolved with neurological and digestive symptoms in the context of multiple secondary determinations and, subsequently, exitus [49]. Since then, multiple cases of vulvar and vaginal melanomas with various clinical, dermatoscopic aspects, and symptoms have been depicted in the literature (for additional details see [16,20]).

As it is a rare pathology, most of the clinical data presented in the literature are obtained from isolated case presentations or case series, most of which highlight the presence of genital bleeding, a palpable, white-gray color mass, itching, dyspareunia, yellow genital secretions, and local pain [50,51,52,53,54]. Cases that mention the presence of painless masses are also reported [55]. Most of the cases presented depict the tumor in the labia minora. The diagnosis is generally late, with half of the patients being diagnosed in advanced stages, with the invasion of deep structures [56].

Although not so numerous and generally on small patient samples, there are prospective or retrospective clinical studies and meta-analyses that have highlighted the main clinical manifestations and the most common sites of vulvar and vaginal melanomas, which are further illustrated in Table 1.

Regarding the dermatoscopic examination, it is characterized by asymmetrical, irregular aspects, both in terms of contour and pigmentation, lacking a clear structure. In addition, multiple cases report the presence of blue-gray or white-gray areas and globular punctiform black-brown structures [45,58].

## 4. Histological Aspects

Melanoma may arise de novo or within an existing benign or dysplastic nevus. Lesions usually measure between 2–4 mm in thickness and are frequently ulcerated. In addition to this clinical aspect that raises the suspicion of a malignant lesion, histopathological examination is absolutely necessary to establish the diagnosis.

### 4.1. Macroscopic Examination

The distribution of melanomas on the skin can be at any level, therefore areas not exposed to the sun should not be neglected, which is practically the case of vulvar melanomas. Considering that vulvar melanomas often appear in older women, they should be advised to self-examine with a hand-held mirror [13]. The first step in examining pigmented nevi, moles, and brown spots and growths is to apply the ABCDEs rule and the Ugly Duckling sign (although not applicable for vulvar melanomas) [61]. As for the rest of the skin, vulvar melanomas are black or dark brown, but may also vary from white, pink, red, to other colors [13,62,63].

**Figure 2:**
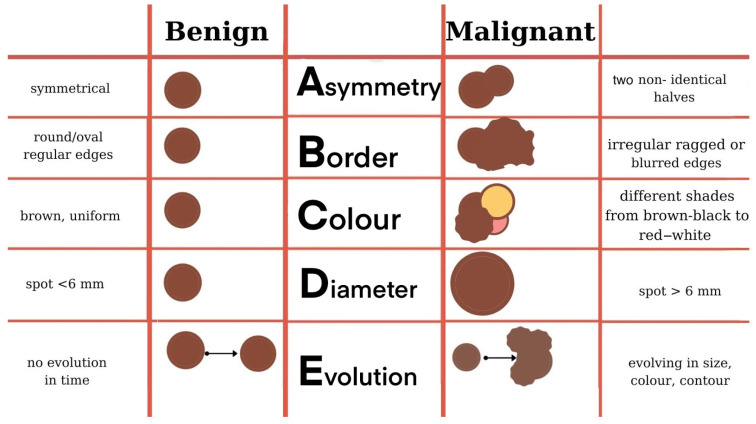
ABCDE evaluation of a pigmentary skin lesion [64].

The ABCDE rule refers to the main macroscopical aspects taken into consideration when assessing cutaneous lesions, namely Asymmetry (the two halves of the mole are not identical), Border irregularity (edges are ragged or notched), Color variation (shades of tan, brown, or black and sometimes patches of red, blue, or white), Diameter (>6 mm or >1/4 inch) or Dermoscopic structure, and Evolution (Figure 2) [64]. The same rule can be regarded from a histological point of view, thus becoming the ABCDE(FG) rule, which refers to Asymmetry (silhouette and color imbalance), Buckshot scatter (pagetoid distribution), Cytological atypia, Deep mitosis, Enclosing lymphocytes, Fibrosis, and Gainsaying (=no) maturation [65].

Another step in the diagnosis of vulvar melanoma is dermoscopy, which facilitates early detection based on the identification of a lesion with irregular dots, multiple colors (black, blue, brown, pink, gray, and white), a blue-white veil, and atypical vessels [66]. When the lesion is assessed and determined as suspicious, the next step is to determine the most important factor for the future evolution and prognosis—the vertical depth of invasion [67]. Therefore, the most effective way to assess the local invasion is the sampling of the lesion. The evolution of melanoma depends on the correctness and accuracy of the biopsy technique. Thus, two types of biopsies of suspicious formations are described: an excisional biopsy that removes the entire lesion, and an incisional biopsy, which removes only a portion from the suspicious cutaneous lesion [68]. Excision biopsy is considered the gold standard in the correct diagnosis of melanomas, and has the advantage of providing accurate microstaging [69].

The 7th edition of The American Joint Committee on Cancer (AJCC) Cancer Staging Manual published in 2009 was the first to give up the Clark score, which was largely based on the level of anatomical invasion in the skin layers [70]. Further on, the 8th AJCC edition on melanoma staging and classification relies on the Breslow thickness in order to establish the depth of invasion, so as to accurately assign a stage based on the classical tumor, node, metastasis (TNM) scores [71]. Additionally, this latest edition specifies that tumor depth should be measured to the nearest 0.1 mm instead of 0.01 mm, in order to improve precision [72]. Although there is not yet a standardized and unanimously accepted staging of vuvar melanomas, the Gynecologic Oncology Group (GOG) recommends the use of the AJCC staging manual instead of the International Federation of Gynecology and Obstetrics (FIGO) system, in spite of the several uncertainties that still persist [18]. Specifically, while generally regarded as mucosal tumors, the molecular characteristics of vulvar melanomas differ from those of both cutaneous and mucosal melanomas in terms of mutational signatures, thus prompting some authors to consider them a unique subclass [21,73].

### 4.2. Microscopic Examination

From a cytological point of view, melanoma cells are of two types: epithelioid and spindle cells. The epithelioid type is characterized by large, round cells with abundant eosinophilic cytoplasm. They have vesicular nuclei with coarse irregular chromatin with peripheral condensation (pattern known as “peppered moth” nuclei). This cell type is most commonly found in nodular and superficial spreading melanomas. The stromal compartment is accompanied by a variable inflammatory infiltrate (brisk, non-brisk, absent), irregular distribution of the pigment, and dermal fibrosis.

### 4.3. Histological Subtypes of Melanoma

The class of tumors known as melanoma contains numerous histological subtypes that are not often considered of the utmost importance to clinicians or patients, especially in terms of prognosis [74]. Histopathological characteristics of the gynecological melanomas should be interpreted in the context of other clinical diagnostic criteria and macroscopic data.

Superficial spreading melanoma (SSM) is the most common subtype, characterized by asymmetrical proliferation of atypical melanocytes. Individual melanocyte units are distributed at the dermo-epidermal junction and are characterized by the presence of large pagetoid areas (>0.5 mm^2^) [75].

Lentigo maligna melanoma (LMM) is characteristic for elderly patients repeatedly exposed to chronic sun damage. Melanocytes arranged in solitary units along the dermo-epidermal junction are organized in small nests (lentiginous pattern), sometimes the nests being horizontally confluent and variable in size and shape (nevoid/dysplastic-like pattern). Dermal invasion of atypical melanocytes is frequent, as well as the extension into the hair follicles [76].

Acral lentiginous melanoma (ALM) is more common in the Afro-Asian population, on palms and soles, and involves the eccrine glands excretory ducts [77].

Nodular melanoma (NM) is considered the most aggressive form, and grows in depth rather than in diameter, so that the ABCDE rule does not apply [78]. It is characterized by a nodular dermal proliferation of atypical melanocytes.

Regarding vulvar melanomas, a retrospective study based on 43 patients, performed by DeMatos et al., showed that most of the mucosal melanoma lesions (vulvar, vaginal, and cervical) were not classified (33%), while the rest were acral lentiginous (30%), or superficial spreading melanoma (26%). Unclassified tumors were either nodular or polypoid melanoma [79].

**Figure 3:**
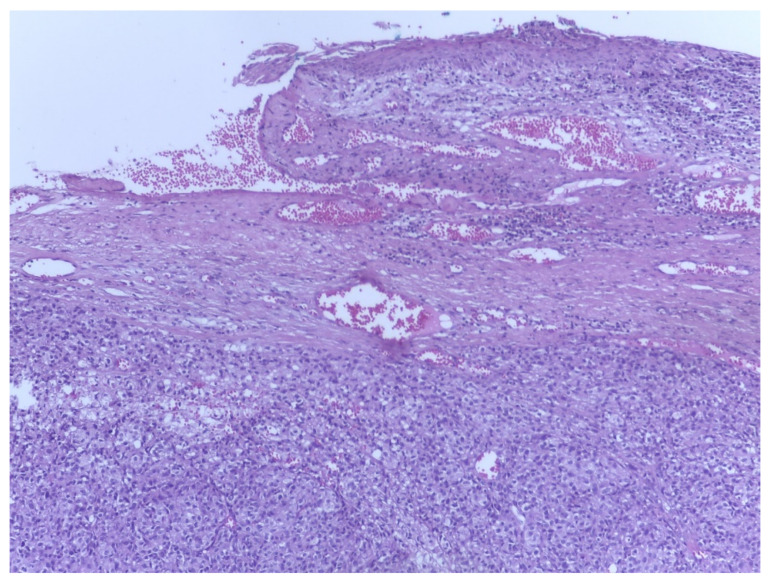
Vaginal melanoma. Vaginal squamous epithelium with areas of ulceration. In lamina propria one can observe tumoral cells organized in nests. Hematoxylin-eosin stain, original magnification ×4 (histological image courtesy of dr. Ileana Popa).

**Figure 4:**
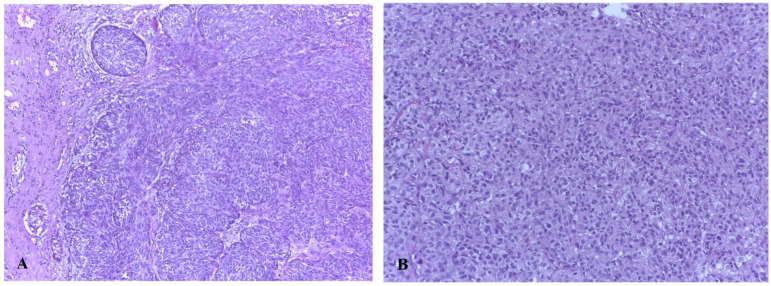
(**A**) Vaginal melanoma. Tumor cells organized in nests, some fusiform-looking cells (Hematoxylin-eosin stain, original magnification ×10.) (**B**) Vaginal melanoma. Epithelioid-looking malignant cells in lamina propria outlooking a nest disposition, abundant eosinophilic cytoplasm. Hematoxylin-eosin stain, original magnification ×20 (histological image courtesy of dr. Ileana Popa).

**Figure 5:**
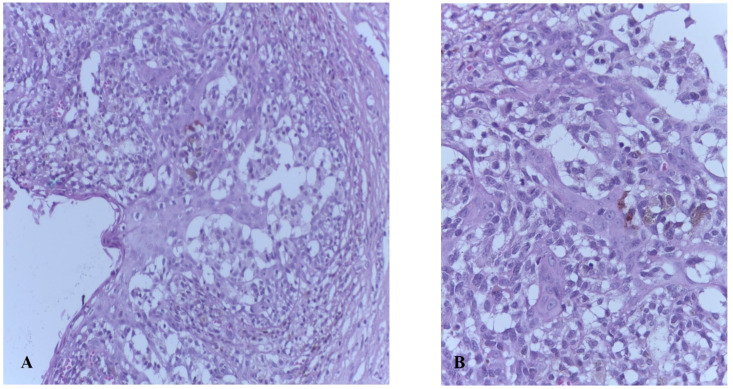
(**A**) Vaginal melanoma. Tumoral cells filled with melanin organized in nests at the epithelium-lamina propria junction—pathognomonic for the diagnosis, original magnification ×20. (**B**) Detail showing haphazardly distributed atypical melanocytes in lamina propria original magnification ×40 Hematoxylin-eosin stain (histological image courtesy of dr. Ileana Popa).

**Figure 6:**
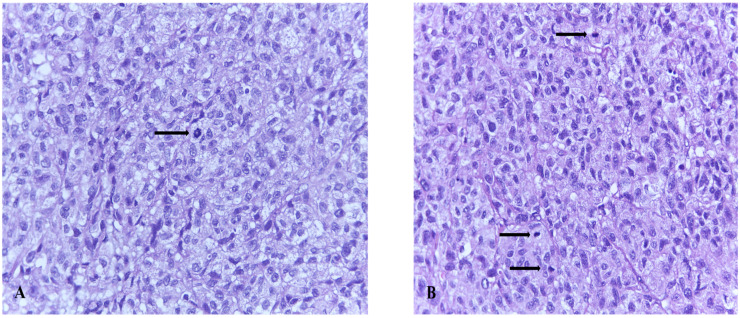
(**A**,**B**) Vaginal melanoma. Arrows—Nuclear mitoses—original magnification ×40.Hematoxylin-eosin stain (histological image courtesy of dr. Ileana Popa).

The most common vulvar melanomas subtypes are mucosal lentiginous and nodular melanoma. The characteristic microscopic aspects are represented by impairment of the epidermis, pagetoid spread of melanocytes, or melanocytes organized in nests (Figure 3 and Figure 4) [80]. Usually, melanocytes are variable in size and shape, and are sometimes localized within lymphovascular spaces. Furthermore, they can frequently become confluent and lack maturation, showing atypical mitoses and increased apoptotic activity (Figure 5 and Figure 6) [81]. The presence of an ulcerated tumor is associated with a poor prognosis.

## 5. Immuno(cyto)histochemistry in the Diagnosis of Melanoma

The criteria described above are imperiled by subjective interpretations, lacking objectivity, and reproducibility. Moreover, melanomas are able to mimic a lot of other malignancies with epithelial, hematologic, mesenchymal, and neural origin, such as lymphomas, carcinomas, neuroendocrine tumors, and sarcomas [82]. Therefore, immunocytochemistry (IHC) is a step most often necessary in the correct evaluation of a melanocytic suspect lesion in patients with underlying hematological pathologies or with solid cancers with frequent skin metastasis. Immunohistochemistry can show its utility in the differential diagnosis of vulvar soft tissue lesions, i.e., Paget disease, which can mimic melanoma [83]. In addition, IHC is less expensive by comparison with electron microscopy, which can bring additional information for melanoma cells containing melanosomes and other ultrastructural particularities which are not present in Paget disease.

Melanomas are usually immunoreactive for Melanoma antigen recognized by T-cells-1 (MART-1) or melan A, S-100 protein, melanoma-specific antigen (HMB-45), tyrosinase, and Microphthalmia transcription factor (MITF) [84].

S-100 protein, a sensitive marker for melanocytic differentiation, is considered to have a sensitivity of 97–100%, but the specificity for melanoma is low since is also expressed on glial cells, Schwann cells, chondrocytes, lipocytes, dendritic cells, histiocytes [85].

One of the first specific markers that was discovered is HMB45, a marker of the cytoplasmic pre melanosomal glycoprotein gp100 [86].

Melan A is the most widely used technique for identifying basal melanocytic proliferations, having a more intense and diffuse staining than HMB45 and a specificity of 95–100% [87].

Further on, the Sry-related HMg-Box gene 10 (SOX10) nuclear transcription factor is a key player in the differentiation of pluripotent neural crest cells into melanocytes. In this regard, its use as a metastatic melanoma marker has been investigated and found to be highly specific and sensitive [88].

## 6. Molecular Characterization

The pathogenesis of genital tract melanomas can mimic that of cutaneous melanoma developed in areas that are sun-protected, as in the case of vulvar melanomas. Moreover, other types of mucosal melanomas (e.g., the respiratory and the gastrointestinal tract) can serve as a model for the development of vaginal or cervical melanomas. Still, there are gaps in understanding the pathogenesis of vulvar or vaginal melanomas. However, elucidating the pathways involved in melanoma genesis is a very important step towards future advanced precision treatment.

Mucosal melanomas represent a challenge in relation to treatment, since, as opposed to cutaneous melanomas, not only are they detected in more advanced stages, but they are also less responsive to immunotherapy and lack activating mutations involving dominant MAP kinases [89,90]. Mutations in KIT, NF1 and SF3B1 genes are more frequently seen in mucosal melanomas, while alterations to NRAS and BRAF genes are more common in cutaneous melanomas [91,92]. Female genital tract melanomas can sometimes contradict the pattern described above. A study involving the whole-genome analysis of 284 patients diagnosed with mucosal melanoma localized in different sites revealed 10 mutated genes (Table 2) [93].

A retrospective study performed by Rouzbachman et al. on 33 vulvar and 11 vaginal malignant melanomas identified by next-generation sequencing analysis, found that the most frequent mutations were in C-KIT and NRAS genes, while BRAF mutations were infrequent. The study did not establish a correlation with the prognosis or outcome for these patients [94].

A more recent study performed by Cai et al. on 19 melanomas of the female genital tract and paired with 25 cutaneous melanomas, 18 acral melanomas and 11 melanomas of the nasal cavity, concluded that malignant melanoma of the female genital tract harbors distinct mutation rates in the KIT, BRAF, SF3B1, KRAS, and NRAS genes. Moreover, this location of the melanoma is an entirely distinct entity from skin melanoma and melanomas of the nasal cavity, while cervix melanomas also have some particularities, and do not suffer recurrent KIT mutations, or mutations of the NRAS and SF3B1 genes [95].

Because BRAF and NRAS mutations are uncommon in non-cutaneous melanomas, we can consider that each melanoma subtype depends on a different oncogenetic pathway for its development. This leads to the idea that it will not benefit from anti-RAF treatment, although large studies are needed before considering this as a rule. However, in the era of precision medicine, the importance of driver mutations cannot be neglected, and should become part of mucosal melanoma routine clinical testing [96].

**Table 2:** The main mutations that occur in melanomas and their frequency of occurrence [93,97,98,99,100,101,102].

Mutated Gene	Percentage of Mutation (%)	Details
NRAS	12/67—17.9%	codon 61—less involved in mucosal melanomas codon 12—more involved in mucosal melanomas more frequent in case of metastatic or recurrent melanoma [93]
BRAF	11/67—16.4%	mostly mutations in protein tyrosine kinase domain predominant targeting the hotspot region between amino-acids 594 and 600 [93]
NF1	11/67—16.4%	are correlated with melanomas developed on high UV exposure areas [97] NF-1 mutation melanomas are more frequently involving female patients, have a higher Breslow score, and can be associated with subsequent neoplasia [98]
KIT	10/67—14.9%	are frequently screened for mutations in exons 9, 11, 13, 17, and 18 KIT mutations are more frequently involved in vulvar melanoma than in other types of mucosal melanomas single substitutions of an amino acid are the most frequent KIT mutations, the most common being L576P [99]
SF3B1	8/67—11.9%	more frequently in European ancestry patients mostly in primary melanomas [93]
TP53	6/67—8.9%	is correlated with response to immunotherapy with CTLA-4 blockade [100]
SPRED1	5/67—7.4%	deletion of the SPRED1 gene leads to developing resistance to MAPK inhibition, so this mutation is essential in evaluating the choice of treatment [101]
ATRX	4/67—5.9%	ATRX works as a chromatin remodeler, thus progression of a melanoma lesion is associated with a reduced expression of ATRX gene [102]
HLA-A	4/67—5.9%	[93]
CHD8	3/67—4.4%	[93]

## 7. Melanoma Stage Description

Detection of a melanoma involving the vulva or vagina associates an essential step: the exclusion of any kind of metastatic dissemination in places such as mucosal membranes, skin, or the eyes [102]. Since staging approved for melanomas, in general, cannot be extended to female genital tract melanomas, there is a great need for dedicated staging.

Clark classification that stages melanomas regarding their level of invasiveness and Breslow classification regarding the vertical depth of the lesion are dedicated for cutaneous melanomas [103,104,105].

A new and modified Clark classification, named Chung classification, is considered specific for micro-staging of mucosal melanomas in general (Table 3) [106].

A 25-year study that both encompasses clinical and pathological features of vulvar melanoma revealed that American Joint Committee on Cancer (AJCC) staging system is the unique prognostic factor for vulvar melanoma (Table 4) [57]. The AJCC system was revised in 2017, associating new prognostic factors [72].

## 8. Therapeutic Approach

Melanomas with vulvar and vaginal localization represent real challenges in terms of treatment, not only due to late diagnosis in locally advanced or metastatic stages, but also due to the absence of specific therapeutic guidelines due to the small number of cases. Thus, due to the impossibility of gathering a significant number of cases to establish dedicated guidelines, the guidelines for cutaneous melanoma are often used.

The main treatment for both vulvar and vaginal melanoma is surgical excision of the tumor. Clinical experience in the last few decades has shown no significant differences in survival rates between radical excision and limited excisions with safety margins (Table 5), so limited resections are currently recommended [32,103]. Pelvic exenteration may be helpful, but only in carefully selected cases [107].

Surgical excision may be completed in some cases by lymphadenectomy. Literature data do not provide specific information on the positive impact of lymphadenectomy in terms of survival, so it is preferred to perform the sentinel node technique in the absence of clinical involvement of regional lymph nodes, followed by lymphadenectomy in the case of a positive result [103]. In the case of clinical invasion of the regional lymph nodes, tumor extension in the adjacent structures, and/or ulceration, lymphadenectomy is recommended, not preceded by the sentinel node technique, which may or may not be followed by adjuvant treatment [108,109]. Lymphadenectomy, although a radical intervention, has no impact on long-term survival, only enabling better local control of the disease [107].

Regarding the utility of radiotherapy, chemotherapy, and immunotherapy, data presented in the literature are limited, but randomized controlled trials show minimal changes in survival [107]. Radiotherapy is generally used as neoadjuvant treatment, in case of impossibility of surgical resection, lymphatic invasion, regional extension, or as palliative treatment [103,110]. Doses of 45–55 Gy are generally used in daily sessions of 1.8–2 Gy/session [103]. The use of radiotherapy in localized forms has been associated with a decrease in survival [110,111].

Chemotherapy is mainly used as a neoadjuvant treatment or unique therapy, utilizing the same agents as those used in skin melanoma: dacarbazine and temozolomide, with dacarbazine being the first Food and Drug Administration-approved drug for melanoma treatment [103,112]. In addition, chemotherapy represents, together with surgery, the last therapeutic resource for the treatment of vulvar melanoma diagnosed in advanced stages [107].

Although on small groups of patients, there are studies showing the effectiveness of chemotherapy compared to the use of high-dose interferon for stage II vulvar melanoma [107]. Moreover, the use of temozolomide-cisplatin combination as adjuvant therapy in stages II-III showed statistically significant benefits over the use of interferon-alpha or surgery in terms of survival (*p* < 0.01) [109]. The carboplatin–paclitaxel–bevacizumab combination has been shown to be effective in decreasing tumor volume, thus allowing surgical resection without the need for further grafting [113]. Chemotherapy is used mainly in regionally advanced forms or with distant metastases, but the benefits are minimal, the use of chemotherapy is generally associated with a decrease in overall survival and recurrence-free survival [110,113]. Up until 2011, only four drugs had been approved for the treatment of melanoma; currently, their number has reached 30, and this increase is based mainly on newly emerged molecules from the class of immunotherapeutics or biological drugs [112].

The class of immunotherapeutics includes molecules that interfere with the immune cascade of carcinogenesis, such as interferon α-2b (IFN α-2b), peginterferon α-2b (PegIFN α-2b), interleukin 2 (IL-2), inhibitor of death protein 1 (PD-L1 blockade)–nivolumab, regulatory T lymphocyte inhibitors, monoclonal antibodies against cytotoxic T cell antigen 4 (CTLA-4)–ipilimumab [112]. These are especially used in advanced stages (stage III-IV) of cutaneous melanoma, but for vulvar melanoma, there are not enough clinical data to demonstrate efficacy in terms of overall survival or disease-free survival. However, their use doubled in the period 2012–2015 compared to the corresponding previous period (*p* < 0.01), most frequently for regionally advanced forms and those with distant metastases [110]. IFN α-2b appeared to lead to an increase in disease-free survival in a randomized clinical trial, while no effects on survival after the use of IL-2 alone or in combination with peptide vaccines were observed [107]. Albert et al. observed in a study on 1917 patients with vulvar melanoma (3/4 with localized disease) that the combination of immunotherapy with local surgery was associated with an increase in overall survival, although statistically insignificant [110]. On the other hand, the use of immunotherapy in combination with chemotherapy and biological therapy was associated with a decrease in overall survival and recurrence-free survival in a study on 48 patients with vulvar and vaginal melanomas [113]. The same data were supported by Tcheung et al. who did not find significant differences in overall survival in patients treated with immunotherapy and chemotherapy [114].

Although targeted therapy has evolved in the last years and significantly improved the prognosis of patients with cutaneous melanoma, its use in vulvar and vaginal melanomas is still limited by the different mutagenic profiles involving fewer BRAF mutations and more c-KIT, NF1 and SF3B1 mutations [91,112]. Thus, monoclonal antibodies such as imatinib, sunitinib, dasatinib, nilotinib (for c-kit mutations) could have been a promising option due to a large number of c-kit mutations, but the results are not as good as in the case of cutaneous melanoma. Imatinib has been shown to reduce tumor size and PET uptake, as well as the disappearance of metastases in a patient with recurrent vulvar melanoma and c-kit mutation in exon 13 [115]. There are phase II studies with promising results for the use of imatinib and dasatinib in patients with mucosal melanomas with c-kit mutations [107,116]. Cocorocchio et al. also mention a positive response to avapritinib therapy in a patient with advanced vulvar melanoma with a c-kit mutation in exon 17, who had a previous negative response to the ipilimumab–nivolumab combination [117].

The prognosis is generally poor, characterized by frequent recurrences (up to 70%), with a low overall survival (average survival between 15–78 months), and with a recurrence disease-free interval of up to 64 months [113,114,118,119,120,121] (Table 6). The main prognostic factors are tumor size, lymphatic invasion, Breslow depth, and the presence of ulcerated lesions [114,122].

Vaginal melanoma is characterized by higher recurrence rates and lower average survival, requiring wider excisions with complete lymphadenectomy and even pelvic exenteration [113,121,123,124]. Along with the surgical excision, adjuvant chemotherapy (dacarbazine) or the combination of targeted therapy (ipilimumab)–radiotherapy have been mentioned in the literature in isolated case presentations or case series, with favorable results [113,121]. In the case of the combination of ipilimumab–radiotherapy, it was found that the administration of radiotherapy in doses of 3000 cGy in 5 fractions was accompanied by the absence of recurrences compared to radiotherapy in doses of 6020 cGy in 28 fractions (for isolated cases, no statistical significance was demonstrated in randomized clinical trials) [113].

## 9. Conclusions

Vulvar and vaginal melanomas are rare neoplasms, with aggressive evolution, most often diagnosed in advanced stages. Gynecological melanomas currently represent a discouraging diagnosis due to the absence of specific therapeutic guidelines and management measures with satisfactory results. Despite the significant development of targeted therapy that has brought major benefits in the prognosis of cutaneous melanoma, vulvar and vaginal melanoma have a low response to these therapies due to the presence of different mutagenic profiles. Thus, despite the use in various clinical trials of many types of targeted therapies, or combinations of chemotherapy and immunotherapy, surgical excision within safe limits remains the therapeutic approach with the best survival rate. The molecular mechanisms of the vulvar and vaginal melanomas are not yet fully understood, these types of melanomas being considered an independent subcategory of melanoma. As new mechanisms are discovered, new targeted therapies may appear to improve the prognosis and subsequently, the survival rates.

## Figures and Tables

**Table 1:** Clinical findings in melanoma of the vulva and vagina [15,26,36,47,57,58,59,60].

Number of Patients	Tumor Localisation	Main Signs and Symptoms	Others Signs and Symptoms	References
51	Labia minora	Pain, Palpable mass, Genital bleeding, Pruritus	Dysuria, Ulceration	[57]
20	Labia majora	Genital bleeding, Pruritus, Palpable mass	Pain, Dysuria, Unhealing sore, Urinary difficulties	[36]
10	Labia majora	Pruritus	-	[47]
11	Not specified	Pruritus, Pain, Genital bleeding	-	[26]
14	Labia minora	Pruritus	-	[58]
31	Vagina	Genital bleeding, Pain, Palpable mass	Abnormal vaginal secretion, Urinary difficulties	[59]
33	Not specified	Palpable mass, Genital bleeding, Pain, Pruritus	Abnormal vaginal secretion, Dysuria, Dyspareunia, Ulceration	[60]
198	Unilateral, Clitoris	Genital bleeding, Pain, Pruritus	-	[15]

**Table 3:** Clark and Chung classifications comparative aspects [104,105,106].

Level of Invasion	Clark Classification—Level of Invasion of Cutaneous Melanoma	Chung Classification—Level of Invasion of Mucosal Melanoma
**I**	Lesions involving only the epidermis (in situ melanoma)	Tumor confined to the epithelium
**II**	Invasion of the papillary dermis—does not reach the papillary-reticular dermal interface	Tumor penetrates the basement membrane and invades at a depth of <1 mm
**III**	Invasion fills and expands the papillary dermis but does not extend to reticular dermis	Tumor invades at a depth of 1–2 mm
**IV**	Invasion into the reticular dermis but not into the subcutaneous tissue	Tumor invades at a depth of >2 mm, but without reaching the subcutaneous fat
**V**	Invasion through the reticular dermis into the subcutaneous tissue	Tumor penetrates the subcutaneous fat

**Table 4:** American Joint Committee—prognostic stage group for melanoma 2017 [57,72].

STAGE	T (Tumor)	N (Nodules)	M (Metastasis)
**0**	Tis	N0	M0
**IA**	T1a	N0	M0
**IB**	T1b, T2a	N0	M0
**IIA**	T2b, T3a	N0	M0
**IIB**	T3b, T4a	N0	M0
**IIC**	T4b	N0	M0
**IIIA**	T1a/b, T2a	N1, N2a	M0
**IIIB**	T0	N1b, N1c	M0
	T1a/b, T2a	N1b/c, N2b	M0
	T2b, T3a	N1a-N2b	M0
**IIIC**	T0	N2b/c, N3b/c	M0
	T1a-T3a	N2c, N3	M0
	T3b, T4a	N ≥ N1	M0
	T4b	N1a-N2c	M0
**IIID**	T4b	N3	M0
**IV**	Any T, Tis	Any N	M1

**Table 5:** Safety surgical margins in limited resection of vulvar and vaginal melanomas [15,103,107].

Tumor Width	Margins	Reference
<2 mm	0.5 cm	[103]
2 mm	1 cm	
>2 mm	2 cm	
<1 mm	1 cm	[98]
1–4 mm	2 cm	
invasion of subcutaneous fat/fascia, any size	>1 cm	
in situ	0.5 cm	[15]
<2 mm	1 cm	
>2 mm	2 cm	

**Table 6:** Survival rates according to type of treatment [110,114,115,116,118].

Number of Patients	Mean Depth Invasion	Management	Median Survival (Months)	Reccurence	Reference
13	Not provided	Not provided	15	Not provided	[119]
7	8 mm	Wide local excision	31	71%	[118]
14	3.23 mm	13% Radical surgery and lymphadenectomy 27% Wide local excision and lymph node evaluation 53% Wide local excision	Not provided	42%	[122]
9	4 mm	Radical surgery and lymphadenectomy	78	32–43% (in situ)	[120]
85	3.2 mm	12.9% Chemotherapy 24.7% Immunotherapy (IL-2, IFN, vaccine trials) 2.35% Chemotherapy and Immunotherapy 15.29% Radiotherapy 78.8% Surgery	62.4	Not provided	[114]
48	>3 mm	29.58% Wide local excision and lymphadenectomy 21.42% Radical surgery (8% pelvic exenteration)	39.6	Not provided	[113]
1917	Not provided	95.044% Surgery 10.38% Radiation 9.99% Immunotherapy 5.32% Chemotherapy	Not provided	Not provided	[110]
